# Mechanisms of lactylation modification in hepatocellular carcinoma treatment resistance

**DOI:** 10.1093/gastro/goag003

**Published:** 2026-02-11

**Authors:** Yinan Zhu, Ziyue Wang, Haiyan Xi, Wanchen Lu, Mingfang Sun, Xuyong Lin

**Affiliations:** Department of Pathology, The First Affiliated Hospital and College of Basic Medical Sciences, China Medical University, Shenyang, Liaoning, P.R. China; Department of Pathology, The First Affiliated Hospital and College of Basic Medical Sciences, China Medical University, Shenyang, Liaoning, P.R. China; Department of Pathology, The First Affiliated Hospital and College of Basic Medical Sciences, China Medical University, Shenyang, Liaoning, P.R. China; Department of Pathology, The First Affiliated Hospital and College of Basic Medical Sciences, China Medical University, Shenyang, Liaoning, P.R. China; Department of Pathology, The First Affiliated Hospital and College of Basic Medical Sciences, China Medical University, Shenyang, Liaoning, P.R. China; Department of Pathology, The First Affiliated Hospital and College of Basic Medical Sciences, China Medical University, Shenyang, Liaoning, P.R. China

**Keywords:** hepatocellular carcinoma, lactylation modification, treatment resistance, prognosis

## Abstract

Hepatocellular carcinoma (HCC) has high global morbidity and mortality. Advanced HCC depends on systemic therapies, but primary/acquired drug resistance severely limits patient survival, creating an urgent need for new targets. This review focuses on how lactylation modification drives HCC drug resistance. In recent years, lactylation, a novel type of post-translational modification (PTM) of proteins mediated by the metabolic product lactate, has been found to be widely involved in the regulation of malignant progression, maintenance of stem cell characteristics, and treatment resistance in HCC. Lactylation conjugates lactate to histones and non-histones, regulating gene expression. Key resistance pathways include: lactylated IGF2BP3 activating PCK2-NRF2 to counter lenvatinib-induced stress; ALDOA lactylation enhancing liver cancer stem cell self-renewal for chemoresistance; MOESIN lactylation in Regulatory T cells (Tregs) weakening anti-PD-1 efficacy. HCC lactylation levels are higher than normal tissues (correlating with poor prognosis); lactylation-related genes/models predict treatment responses. Therapeutically, 2-DG, AZD3965, or SIRT3 activators (reverse lactylation) restore drug sensitivity, alone or in combination. Despite limited specific detectors, lactylation is a promising target to overcome HCC drug resistance, aiding precision treatment.

## Introduction

HCC is one of the malignant tumors with the highest incidence and mortality rates, remaining a global public health challenge worldwide [1]. Chronic hepatitis B virus infection and long-term excessive alcohol consumption are currently recognized as the main causes of HCC. However, due to the absence of symptoms in the early stage of HCC, obvious clinical symptoms often lead to delayed diagnosis. For advanced liver cancer (defined as the presence of vascular invasion or extrahepatic metastasis and/or mild cancer-related symptoms), first-line treatment should involve systemic therapies, including chemotherapy, targeted therapy, and immunotherapy [2]. In contrast, surgical resection, local embolization, and interventional ablation are often ineffective for advanced-stage cancer [3]. Unfortunately, most advanced HCC patients do not experience long-term benefits due to primary or acquired drug resistance, and the rising incidence of the disease undoubtedly further amplifies its clinical impact [4, 5]. Therefore, there is an urgent need for novel therapeutic approaches to improve patient prognosis.

As the end product of glycolysis, lactate has long been regarded as a cellular metabolic waste of cells under hypoxic conditions. Its significance in the development of cancer-promoting environments—first highlighted by the Warburg effect in 1956, which proposed the notable role of lactate in cancer progression—was not widely recognized until its importance as a byproduct of high glycolytic rates became evident [6]. The Warburg effect thus underscored that lactate production is increasingly gaining attention as a potential key target for cancer therapy. Studies have indicated that higher levels of lactate may indicate poor prognosis in HCC, making the lactate pathway more significant in the metabolic processes of HCC [7–9]. However, current therapeutic targeting of the Warburg effect remains immature, with preclinical and clinical trials not yet widely carried out or translated into clinical practice [10–12].

**Figure 1.**
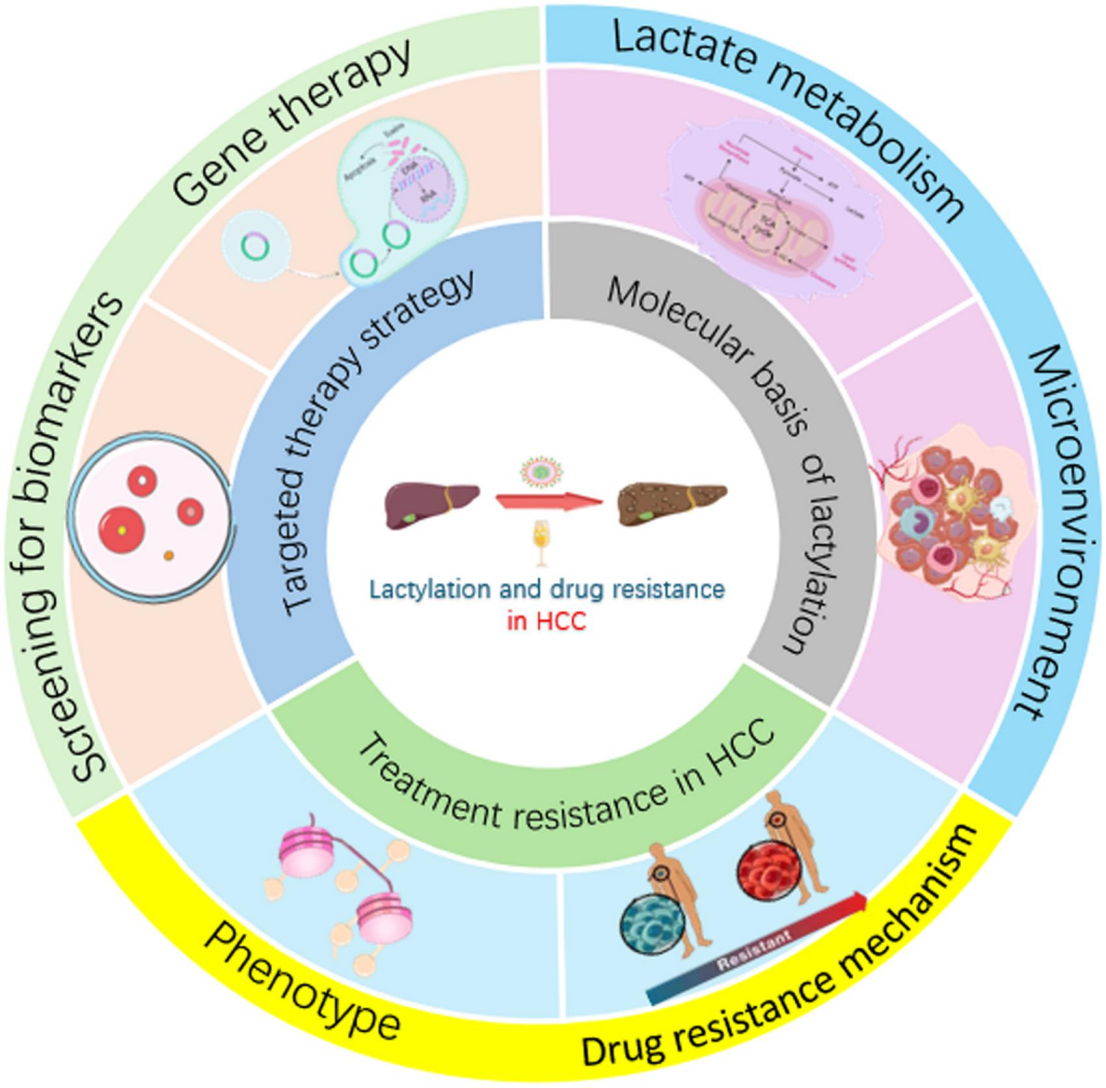
The role of lactylation modification in the treatment of liver cancer. By systematically analyzing the process of lactic acid metabolism, we have comprehensively sorted out the mechanisms underlying drug resistance formation and identified novel therapeutic targets. Lactylation modification significantly influences the immune microenvironment, playing a critical role in the development of drug resistance. In recent years, relevant drug trials have been ongoing. However, there remains a scarcity of drugs that have been successfully translated into clinical applications.

Lactylation is a novel PTM of proteins mediated by the metabolite lactate. By covalently conjugating lactate to the lysine residues of proteins, lactylation regulates gene expression and cellular metabolism, thereby influencing tumor progression, metastasis, and drug resistance [13–15]. Unlike traditional protein modification processes, the role of lactylation in tumor metabolic reprogramming and epigenetic regulation has been gradually revealed. Through modulating the functions of histones and non-histone proteins, this modification process plays a critical role in the initiation, progression, and treatment resistance of HCC [16]. Lactylation has been found to be widely involved in the regulation of malignant progression, stem cell property maintenance, and treatment resistance in HCC [17, 18]. Recent studies have demonstrated that lactylation levels in HCC tissues are significantly higher than those in normal liver tissues and are closely associated with tumor cell proliferation, migration, and immune microenvironment remodeling [19, 20]. This review systematically summarizes the molecular mechanisms underlying lactylation in treatment resistance of HCC and discusses potential therapeutic strategies based on lactylation regulation (Figure 1).

## The Warburg effect in HCC

**Figure 2.**
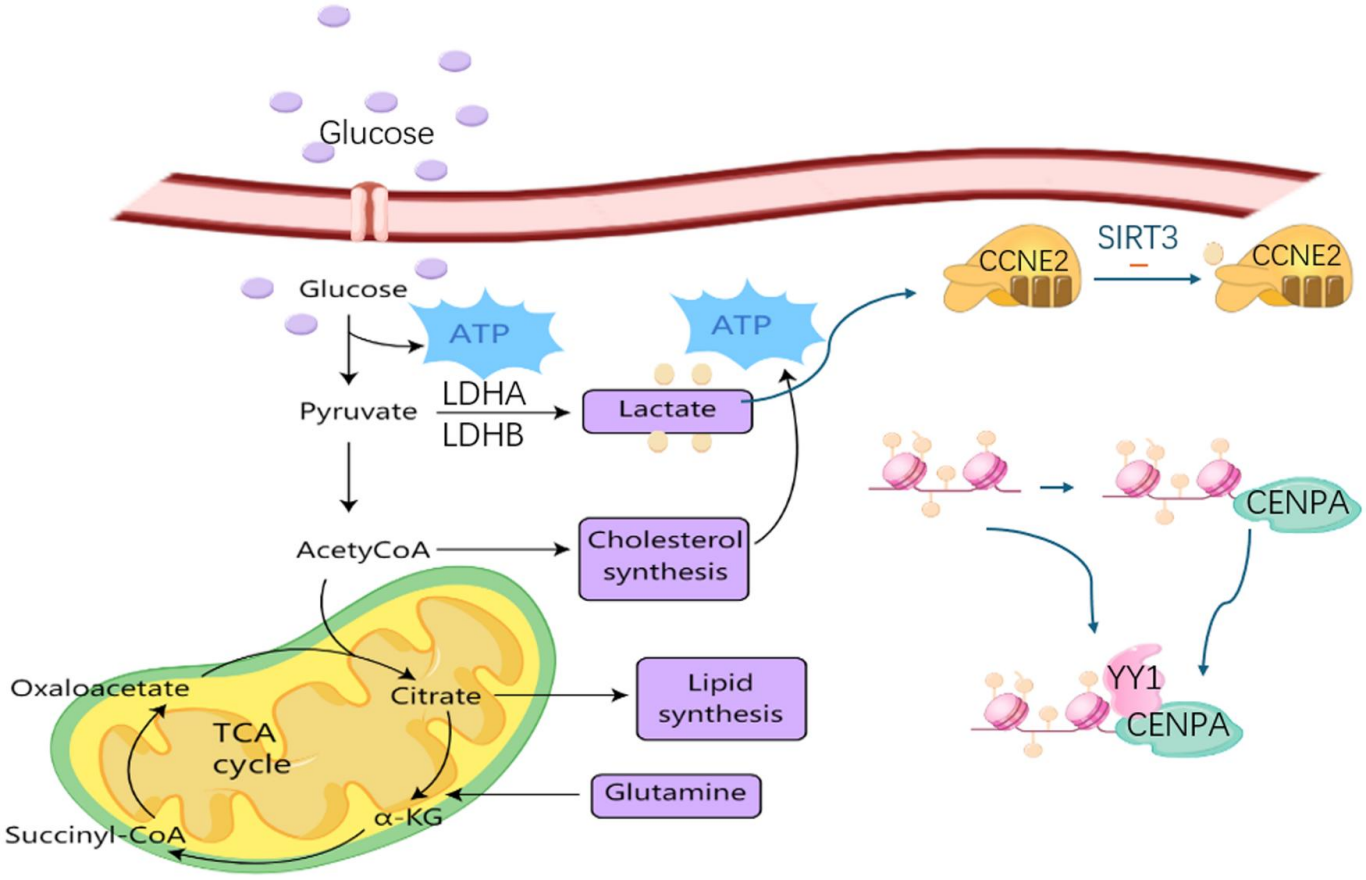
Regulation of lactate production. The dynamic balance of lactylation is bidirectionally regulated by LDHA/LDHB and delactylases (such as SIRT3). In HCC, the hypoxic characteristics of the tumor microenvironment and the Warburg effect lead to massive intracellular lactate accumulation, providing a substrate basis for lactylation. Lactylation can synergize with YY1 through the CENPA-YY1-CCND1/NRP2 axis to promote HCC progression. Studies have shown that lactylation levels in HCC tissues are significantly higher than those in normal liver tissues, and are negatively correlated with tumor grading and patient prognosis. Previous researchers have described this biological process in detail.[31] SIRT3=Sirtuin 3, YY1=Yin Yang 1, CENPA=Centromere Protein A, CCND1=Cyclin D1, NRP2=Neuropilin 2.

The Warburg effect enables tumor cells to generate lactate at a rate 10 times higher than normal tissues in aerobic environments, with lactate serving as an energy substrate [21]. Similar to normal cellular metabolic pathways, any process that promotes pyruvate production in tumor cells enhances lactate generation and ultimately increases lactate efflux. Lactylation, a dynamic modification dependent on lactate concentration, requires lactate to bind covalently to lysine residues of protein. Lactate significantly influences the biological processes of multiple tumor cell types, and animal models have confirmed that reducing lactylation alleviates tumor progression patterns [22–25]. In the tumor microenvironment (TME) of HCC, HCC cells enhance the Warburg effect to produce large quantities of lactate. Beyond altering cellular metabolic states, chromatin structure and gene expression are also affected by lactylation, indicating its significant role in HCC cell proliferation, invasion, and metastasis—and highlighting the promising prospects of glycolysis-based therapies for HCC [26, 27]. In HCC, PDHX acetylation disrupts pyruvate dehydrogenase complex (PDC) assembly and activates lactylation at the histone H3K56 site, ultimately driving HCC progression [28]. Lactylation arises from the covalent conjugation of the carboxyl group of lactate to the ε-amino group of protein lysine residues, with its dynamic balance regulated bidirectionally by lactate dehydrogenases (LDHA/LDHB) and delactylases (e.g. SIRT3) [29]. In HCC, the hypoxic characteristics of the TME and the Warburg effect lead to substantial intracellular lactate accumulation, providing a substrate basis for lactylation [30]. CENPA can be lactylated at lysine 124 (K124), cooperating with YY1 through the CENPA-YY1-CCND1/NRP2 axis to promote HCC development [31] (Figure 2). Studies have shown that lactylation levels in HCC tissues are significantly higher than those in normal liver tissues and negatively correlated with tumor grade and patient prognosis [32, 33].

## Lactylation sites in HCC

**Figure 3.**
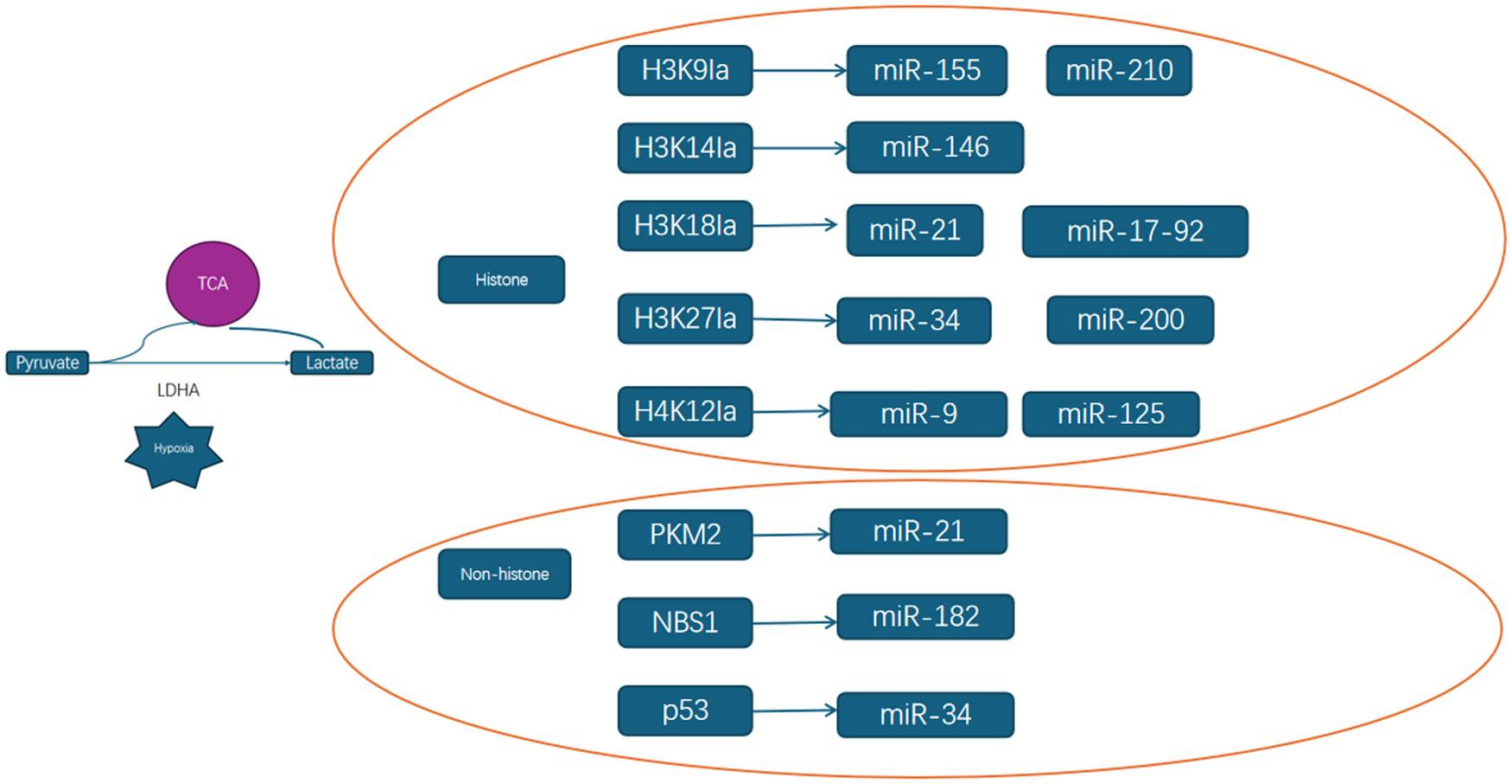
Regulated sites in lactylation process. During the lactylation process, regulated sites are categorized into histones and non-histones. Correspondingly, we have summarized the miRNA regulatory pathways associated with these distinct sites.

Lactylation modifies epigenetic mechanisms to shape the malignant phenotype of HCC. We systematically summarized the microRNA regulatory pathways of histone and non-histone sites in the process of lactylation modification (Figure 3). Multiple studies using mass spectrometry and antibody-specific detection have identified two key upregulated histone lactylation sites in HCC tissues: H3K9la and H3K56la. These modifications induce chromatin structural changes and oncogene transcriptional activation, thereby promoting tumor cell proliferation and metastasis [34–36]. This process is mediated by endothelial cell-specific molecule 1 (ESM1), and inhibition of histone lactylation using 2-deoxy-d-glucose (2-DG) reduces lactylation products, reversing the epithelial–mesenchymal transition (EMT) process. Lactylation of histone H2B at K58 by lactate dehydrogenase A (LDHA) promotes HCC metastasis by inhibiting cellular senescence[37], while ABCF1-K430la drives glycolytic reprogramming in HCC through activation of the KDM3A–H3K9me2–HIF1A axis [38]. Additionally, lactylation is not limited to histones but also targets metabolic enzymes. For example, lactylation of adenylate kinase 2 (AK2) at K28 inhibits its activity, enhancing metabolic adaptability and invasive capacity of HCC cells and further exacerbating metabolic disorders [39]. The significant role of lactylation in non-histone targets cannot be ignored. Hypoxia-driven tumor growth in HCC is directly influenced by ALDOA-mediated lactate levels, and ALDOA gene knockout in HCC cells reduces lactate levels, thereby attenuating ALDOA-deficiency-mediated growth inhibition [40]. Lactylation of ALDOA at K230/322 promotes self-renewal and drug resistance of liver cancer stem cells (LCSCs) by regulating DDX17 function [36]. Hypoxia increases glypican-3 (GPC3) expression in HCC cells, which enhances c-myc lactylation [41].

## Interplay between metabolic features and lactylation in HCC

Lactylation remodels metabolic networks of amino acids, fatty acids, and nucleotides by regulating tricarboxylic acid (TCA) cycle-related enzyme activities, providing biosynthetic precursors for tumor cells [42]. Metabolic hallmarks of HCC cells include enhanced glycolytic flux and suppressed mitochondrial oxidative phosphorylation, processes epigenetically regulated by lactylation. The elevated glycolysis and lactate accumulation in HCC provide a substrate basis for lactylation, which further exacerbates metabolic reprogramming by modulating metabolic enzyme activities. Apart from H3K56 lactylation [28], H3K18 also plays a vital role in HCC process. At another lactylation site, H3K18 lactylation upregulates glucose transporter 1 (GLUT1) expression, driving metabolic reprogramming via DLAT and increasing metastatic potential in HCC cells [43]. During HCC metabolism, the HIF-1α signaling pathway enhances glycolysis and lactate production. Genes such as LYRM2, TRPM7, and C1R further promote HCC progression by augmenting HIF-1α-dependent metabolic reprogramming [44–46]. In HCC, the lactylation-driven IGF2BP3-PCK2-SAM-m6A loop maintains elevated PCK2 and NRF2 levels, sustaining N6-methyladenosine (m6A) methylation and promoting lenvatinib resistance [47].

## Effects of lactylation on the immune microenvironment in HCC

Histone lactylation not only explains tumor metabolism in cancer patients but also offers potential as a therapeutic target for tumors [48]. Lactate accumulation drives tumor progression by impairing immune cell function, recruiting immunosuppressive cells as signaling molecules, and regulating immune checkpoint pathways. The PD-1/PD-L1 costimulatory pathway plays a role in this process [49]. Lactylation contributes to immune evasion in HCC by regulating immune cell functions. Studies have found that elevated lactylation levels are associated with the activation of cancer-associated fibroblasts (CAFs) and the infiltration of immunosuppressive cells (such as Treg cells) [50, 51]. Additionally, histone lactylation affects the tumor-infiltrating ability of natural killer (NK) cells by regulating the expression of chemokines (e.g. CCL5). Notable progress has been made in investigating lactate levels within tumors and TME, with key findings summarized in Table 1.

**Table 1.** The role of lactate in immune TME components

Cell type	Regulatory mechanism	Biological effect	References
CD4+ T cells	Directly inhibit T cell-mediated immune response	↓ CTL function	[52–58]
	Inhibit TCR activation and reduce induction of T cell apoptosis	↓ Anti-tumor immunity	
	Inhibition of p38 and JNK/cJun signaling proteins	↓ T cell proliferation	
	Enhanced PD1/PD-L1 pathway	↓IFN-γ, ↓TNF-α	
	Promote Th17 cell differentiation	↓NAD+levels	
	Accelerate the redox state of NAD (H)	↑ IL-17, ↓ IL-2	
	Acidic pH TME		
CD8+ T cells	Directly inhibit T cell-mediated immune response	↓ CTL function	[59–63]
	Reduced NAD utilization and cellular motility	↓ Anti-tumor immune activity	
	Inhibit the differentiation of monocytes and dendritic cells	↓ T cell proliferation	
	Inhibition of JAK-JUN pathway and production of cytotoxic cytokines	↓ IFN-γ, TNF-α, and IL-2	
	Enhanced PD-1/PD-L1 pathway		
	Inducing CTL inactivation		
	Acidic pH TME		
Macrophages	Reduce NF–κB activation	↑ Glycolysis	[64–69]
	Reduce the secretion of cytotoxic cytokines	↑Release of TNF-α	
	Enhance the ERK-STAT3 pathway, GPR132, and Notch pathway;	↑IL-23/IL-17	
	Enhance HIF-1a stabilization;	↓ M1 effect ↑ M2 effect	
	Inducing TAM transformation into M2 polarized cells		
	Preventing lactate secretion in macrophage precursors,		
	Inhibition of anti-tumor Th1 response		
Dendric cells	Inhibiting the differentiation of monocytes into dendritic cells,	↑ IL-10 , ↓ IL12, ↓ IL-6	[70–74]
	Cytokine inactivation and transformation into drug-resistant phenotype	↓ IFN-γ	
	Inhibiting the glycolysis process by interfering with the energy generation pathway of pDC		
	Enhance GPR81 signal; reduce TLR signaling		
Natural killer cells	Impairment of NK cell activation and metabolic function	↓ IFN-γ	[75–79]
	Can prevent the activation of nuclear factor activated T cells (NFAT) in NK cells,	↑TGF-β	
	Reduce NFAT activity	↑IL-10	
	Reduce NKp46 activity	↑Number of MDSCs	
	Inhibition of mTOR signaling pathway	↑ Cell apoptosis	
	Acidic pH TME		
Myeloid-derived suppressor cells	Enhance cell secretion	↑Secreted G-CSF and GM-CSF	[80–82]
	Promote the growth of bone marrow mesenchymal stem cells		
	Enhance immunosuppressive effect		
T-regs	Upregulation of P3 protein (FoxP3) expression	↑Differentiation and proliferation of T-reg	[83–85]
	Promote the polarization and activation of Tregs		
	MCT1 mediated lactate influx and intracellular lactate metabolism		

## Role of lactylation in treatment resistance of HCC

A glycolysis-lactylation positive feedback loop has been identified in lenvatinib-resistant HCC models. In this loop, cancer cells upregulate glycolytic activity to increase lactate production, which promotes lactylation of IGF2BP3. Lactylated IGF2BP3 enhances the stability of PCK2 mRNA by binding to its m6A-modified sites, thereby activating the PCK2-NRF2 antioxidant pathway to counteract lenvatinib-induced oxidative stress. Additionally, lactylation of ALDOA at K230/322 dissociates the DDX17 complex, a mechanism that enhances the self-renewal capacity of LCSCs and contributes to chemoresistance [36]. Lactylation also impacts mitochondrial function by regulation of TCA cycle-related enzymes. Lactylation of AK2 at K28 inhibits its enzymatic activity, leading to impaired ATP synthesis. This energy imbalance activates the AMPK/mTOR pathway, which in turn promotes HCC cell proliferation and metastasis [39]. Furthermore, reduced expression of mitochondrial SIRT3 results in accumulated lactylation, a phenomenon that correlates with sorafenib resistance in HCC [86]. Collectively, these lactylation-mediated mechanisms contribute to treatment resistance in HCC, significantly limiting the clinical efficacy of molecularly targeted drugs and chemotherapy regimens.

In HCC drug-resistant cells, elevated levels of H3K9la and H3K56la promote the expression of pro-survival genes GP73 and NDRG1 by relaxing chromatin structure [34, 87, 88]. In oxaliplatin (OXA) and 5-fluorouracil (5-Fu) resistance models, histone lactylation promotes ubiquitination and degradation of PTEN by upregulating NEDD4 expression, which promotes ubiquitination and degradation of PTEN. This process activates the PI3K/AKT pathway, ultimately inhibiting chemotherapy-induced apoptosis [89]. Notably, the 5-hydroxytryptamine receptor 1D (HTR1D) also contributes to resistance to treatments like sorafenib through the PI3K/Akt pathway [90]. The non-coding RNA interaction network is another key target of lactylation regulation, particularly through its effects on RNA-binding proteins. Lactylated IGF2BP3 enhances the invasive capacity of HCC cells by stabilizing MALAT1 lncRNA, a process that involves the promotion of EMT [91]. This aligns with the observation that IGF2BP3, which is upregulated under hypoxic conditions, promotes EMT and augments circRNA biogenesis. Additionally, PYCR1-mediated H3K18la promotes sorafenib resistance in HCC by activating the IRS1/PI3K signaling pathway [92].

Lenvatinib resistance poses a significant challenge in the clinical management of advanced HCC. Multiple intervention strategies targeting lactylation-regulating enzymes or metabolic pathways have entered preclinical research. For example, the glycolytic inhibitor 2-DG reduces intracellular lactate levels and reverses H3K56la-mediated abnormal gene expression, thereby restoring sensitivity to lenvatinib [34]. Additionally, the LMRG model—a patient stratification model based on lactylation modification signatures—combines gene expression profiles such as ACACA and MRPL3 to predict individualized treatment responses [93].

## Vital pathway in lactate production

There are several pathways involved in lactate production. The PI3K/Akt signaling pathway is widely recognized for its promotional role in the initiation and progression of digestive system tumors [18]. As the core molecules of this pathway, the PI3K family consists of three subtypes, each regulating distinct molecular pathways [94]: Class I PI3K generates PIP3, thereby regulating physiological processes such as cell growth and metabolism; Class II PI3K activates the AKT signaling pathway through the synthesis of PIP2; furthermore, Class II and Class III PI3K can jointly produce PI3P, which is involved in the regulation of membrane trafficking and autophagy. PI3K-related signaling pathways have paved the way for the development of PI3K inhibitors [95].

The activation of the PI3K/AKT/HIF-1α signaling pathway is closely associated with enhanced tumor invasiveness. Studies by Wei *et al*. [96] have fully elaborated on the mechanism by which histone lactylation regulates tumors through this pathway in endometrial cancer: on one hand, it upregulates the expression of USP39; on the other hand, USP39 interacts with PGK1 to accelerate the activation of the PI3K/AKT/HIF-1α signaling pathway. Notably, as a key glycolytic enzyme, PGK1, in addition to its traditional metabolic functions, has been confirmed to possess protein kinase activity. It can stabilize HIF-1α through phosphorylation, further activating the expression of downstream genes, thereby promoting the survival and migration of tumor cells in hypoxic environments [97].

mTOR is a key regulatory factor in the PI3K/Akt/mTOR signaling pathway, regulating pathway activity through positive and negative feedback mechanisms [98]. Among them, mTOR can affect tumor invasion and progression through phosphorylation [99]. After mTOR activation, downstream S6K can reduce PI3K activity, and weaken the activity of the PI3K/Akt/mTOR pathway through negative feedback, thereby inhibiting tumor cell activity. Adenosine monophosphate-activated protein kinase (AMPK), as an integrated metabolic sensor in the PI3K/Akt/mTOR signaling pathway, is closely related to tumor development. A study has found that elevated lactic acid levels in HCC can lead to AMPK inactivation, which further accelerates tumor invasion. Therefore, the accumulation of high levels of lactic acid in tumor cells may reduce mTORC1 activity by activating AMPK, indirectly weakening the PI3K/Akt/mTOR signaling pathway, and thus interfering with tumor progression [100].

The activation and regulation of HIF-1α is one of the core mechanisms by which tumors adapt to the microenvironment and also a key downstream effector molecule of the PI3K/AKT pathway [101]. Hypoxia in the tumor microenvironment is an important inducer of HIF-1α activation: hypoxia hinders the hydroxylation of proline on HIF-1α, inhibits the binding of the pVHL tumor suppressor gene, leads to pVHL inactivation, and thereby prevents HIF-1α from being degraded. Other stimulatory factors such as insulin, insulin-like growth factor 1, epidermal growth factor, and angiotensin II can also increase intracellular HIF-1α levels [102, 103].

Upon activation, HIF-1 affects tumor progression primarily through upregulating vascular endothelial growth factor to promote angiogenesis and upregulating erythropoietin to stimulate erythropoiesis, it significantly enhances oxygen and nutrient supply to tumor tissues [104]. In addition, by directly upregulating glucose transporters and indirectly promoting the expression of glycolysis-related enzymes (especially hexokinase, pyruvate dehydrogenase, and lactate dehydrogenase), it enhances the aerobic glycolysis capacity of tumor cells while inhibiting the oxidative phosphorylation pathway—and the lactic acid produced by glycolysis can in turn act on tumor cells through the aforementioned PI3K/AKT/AMPK pathways, forming a ‘metabolism-pathway-microenvironment’ regulatory loop [105].

## Immune microenvironment remodeling and immunotherapy resistance

Tregs employ flexible metabolic strategies to survive in diverse environments, utilizing lactylation as an alternative energy source to fulfill their immunosuppressive functions [106]. Lactate in the tumor microenvironment enhances the immunosuppressive function of Tregs by inducing lactylation of the MOESIN protein, thereby attenuating the efficacy of anti-PD-1 therapy [51, 107]. Clinical data show that MOESIN lactylation levels in Tregs from anti-PD-1-responsive HCC patients are significantly lower than those from resistant patients. As a key energy source for Tregs, increased lactate production drives the activation of PD-1+ Tregs, substantially increasing the likelihood of immunotherapy failure [108].

Tumor-associated macrophages (TAMs), a critical component of the TME, exhibit diverse molecular phenotypes, with M1 and M2 macrophages being representative subtypes [109]. Lactylation sustains the tumor-promoting activity of TAMs [110]. Lactylation promotes M2 polarization of TAMs by activating the HIF1α/IL-10 axis, thereby inhibiting the antitumor activity of CD8+ T cells [111]. LDHA-targeted inhibitors can reverse this immunosuppressive phenotype and enhance the efficacy of immune checkpoint inhibitors [112]. Lenvatinib induces the TME to secrete CXCL2 and CXCL5, promoting neutrophil infiltration and increasing immunosuppressive CD8+ T cells, which hinder its therapeutic effect on tumor cells. Tumor-derived lactate enters neutrophils via MCT1, activating the NF-κB/Cox-2 pathway to induce PD-L1 expression. Lactate and H+ ions prolong neutrophil lifespan and further upregulate PD-L1 [113–115]. Lactate produced by tumor cells inhibits PD-L1 degradation, creating an immunosuppressive TME in PD-1/PD-L1 blockade-resistant tumors. Combination therapy with PD-L1 antibody-drug conjugates (PD-L1-ADC) targeting PD-L1 and the monocarboxylate transporter 1 (MCT-1) inhibitor AZD3965 effectively treats HCC [116].

Applications and therapeutic strategies targeting lactylation Levels of H3K9la and H3K56la in HCC tissues are significantly correlated with patients’ overall survival (OS) and progression-free survival (PFS), suggesting their potential as independent prognostic markers [34]. Single-cell sequencing analysis further reveals that expression heterogeneity of lactylation-related genes (e.g. LDHA, PDHX) reflects tumor clonal evolution, providing a molecular basis for precision medicine [28]. Lactylation-related gene (LRG) risk models constructed based on databases like TCGA (e.g. EP300, HDAC1-3, CA3) effectively predict HCC prognosis [117–119]. Among them, the CA3 gene exerts tumor-suppressive effects by inhibiting lactylation inactivation at the K36 site mediated by SQLE, and its low expression is associated with poor prognosis in HCC [120].

Therapeutic strategies targeting lactylation currently include the following approaches. First, enzyme activity regulation involves developing small-molecule inhibitors of LDHA to block lactate production, while epigenetic interventions can restore chromatin homeostasis by using histone delactylatase activators. Additionally, metabolic microenvironment remodeling is a promising strategy, such as delivering lactate-consuming enzymes to tumor sites via nanocarrier-targeted systems. Drugs targeting lactylation have demonstrated antitumor potential both *in vitro* and *in vivo* [86, 107]. Furthermore, combination therapies targeting lactate metabolism or key lactylation-associated proteins hold promise for overcoming drug resistance in HCC [47, 111].

Glycolytic inhibitors suppress HK-II activity, leading to glycolytic inactivation and reduced extracellular lactate production [121]. 2-Deoxyglucose (2-DG) and LDHA inhibitors reduce lactylation levels by decreasing lactate generation, which significantly restoring lenvatinib sensitivity in preclinical models [34]. Blocking lactate transporters via MCT1/4 inhibitors represents a valuable approach to overcome glycolysis-related drug resistance [122–124]. For example, AZD3965 inhibits lactate efflux from tumor cells, reducing lactate concentration in the TME and thereby attenuating the suppressive effects of lactylation on immune cells [116, 125, 126].

Lactylation transferase inhibitors can block glycolysis and disrupt metabolic pathways in HCC cell proliferation. Small-molecule inhibitors targeting histone lactyltransferases (such as EP300) specifically reduce H3K9la and H3K56la levels, inhibiting the expression of HCC stemness-related genes [127, 128]. Activating SIRT3 enhances its delactylase activity toward non-histone targets (e.g. ABCF1, AK2), reversing lactylation-mediated oncogenic effects [86]. Demethylzeylasteral (DML) suppresses tumorigenicity of LCSCs by activating SIRT3 [120].

Ethylthiolated oil Pickering emulsions effectively encapsulate anti-PD-L1 antibodies, demonstrating significant potential as therapeutic agents for primary and secondary liver cancer [129]. siRNA-based nanoparticles (such as IGF2BP3-targeted liposomes) can specifically knockdown the expression of drug resistance-associated lactylation proteins *in vivo*, while co-delivering glycolytic inhibitors (e.g. 3-BrPA) to produce synergistic antitumor effects [47]. Additionally, the small-molecule inhibitor tubuloside A targeting ABCF1-K430la has exhibited promising anti-HCC activity in preclinical models [38].

Lactylation inhibitors in combination with immune checkpoint blockade significantly reduce lactate production and histone Kla levels. For instance, 2-DG in combination with anti-PD-L1 antibodies enhances antitumor immune responses by decreasing lactylation in Treg cells, significantly prolonging survival in HCC mouse models [34]. Co-administration of histone deacetylase inhibitors (such as vorinostat) and lactylation inhibitors synergistically suppresses epigenetic remodeling in HCC cells, overcoming resistance to the FOLFOX regimen [89]. High expression of MCT1/2 in Tregs contributes to anti-PD-1 resistance in HCC, with lactylation regulation being a critical component. Inhibiting MCTs may further enhance the therapeutic response to PD-1 inhibitors [129–131].

## Future research directions and challenges

In addition to primary HCC, some rare liver tumors are gradually being discovered [132]. With the application of bioinformatics analysis, more biomarkers are used for diagnosis and treatment of patients [133, 134]. Despite significant progress in understanding the mechanisms of lactylation in HCC, several key questions warrant further investigation. First, the role of lactylation in tumor heterogeneity at the single-cell level remains incompletely elucidated. The functional heterogeneity of lactylation across different HCC subtypes also needs systematic characterization [34]. This process reflects the spatiotemporal specificity of dynamic lactylation modifications. Concurrently, cross-omics integration analyses—integrating metabolomics, epigenomics, and proteomics data—can facilitate the construction of comprehensive regulatory networks [68]. Clinical translation bottlenecks limit the translation of basic research findings. Most inhibitors exhibit off-target effects, necessitating the development of more selective compounds. Additionally, the development of highly specific lactylation detection technologies and toxicological assessments for targeted drugs is essential [69]. Presently, the lack of highly sensitive methods for detecting lactylation sites, coupled with limitations in dynamic modification detection technologies, impedes in-depth mechanistic investigations.

## Conclusions

Lactylation, a critical node connecting metabolic abnormalities and epigenetic dysregulation, plays a central role in HCC initiation, metastasis, and drug resistance, providing important directions for understanding tumor heterogeneity and developing novel therapeutic strategies. Targeting lactate-generating enzymes (e.g. LDHA), modifying enzymes, or related signaling pathways holds promise for overcoming current treatment bottlenecks. Dysregulation of stem cell properties, epigenetic remodeling, and immune microenvironment suppression drives drug-resistant phenotypes in HCC. Strategies targeting lactylation generation, modifying enzymes, or developing specific nanodrugs offer new avenues to combat treatment resistance in HCC.

Future studies need to further decipher the specificity of lactylation sites, dynamic regulatory networks, and their interactions with the microenvironment. Meanwhile, exploring the clinical translation potential of lactylation-based biomarkers and targeted drugs—and advancing precision treatment strategies based on lactylation into clinical practice—will be pivotal. Future research should also integrate single-cell sequencing and spatial metabolomics technologies to reveal the spatiotemporal dynamics of lactylation, thereby facilitating the development of personalized treatment strategies.

## Authors’ contributions

Y.Z., M.S., and X.L. conceived the study. Y.Z., Z.W., H.X., and W.L. wrote the initial draft of the manuscript and prepared figures. All authors participated in the revision of manuscript. All authors have read and approved the final article.
